# Retrospective analysis of preoperative serum tumor marker levels and their correlation with pathological characteristics and prognosis in cervical cancer patients

**DOI:** 10.3389/fgene.2026.1724789

**Published:** 2026-01-20

**Authors:** Yue Shen, Min Kang

**Affiliations:** 1 Faculty of Chinese Medicine, Macau University of Science and Technology, Macau, China; 2 Department of Gynecology, Zhuhai Hospital of Integrated Traditional Chinese and Western Medicine, Zhuhai, China

**Keywords:** CA125, CEA, cervical cancer, pathological characteristics, prognosis, SCC-Ag, tumor marker

## Abstract

**Objective:**

To investigate the relationship between the levels of preoperative serum tumor markers SCC-Ag, CEA, and CA125 and pathological characteristics in cervical cancer patients, and to analyze their predictive value for prognosis.

**Methods:**

A retrospective analysis was conducted on clinical data from 82 patients with cervical cancer (cervical cancer group), 98 patients with benign cervical lesions (benign lesion group), and 80 healthy controls (healthy control group). Serum tumor marker levels were compared among the three groups to evaluate their diagnostic value for cervical cancer. Differences in tumor marker levels were assessed among cervical cancer patients with varying TNM stages, differentiation degrees, and pathological types. Pre-treatment tumor marker levels were also compared between patients with different prognoses.

**Results:**

Patients in the cervical cancer group exhibited significantly higher levels of SCC-Ag, CEA, and CA125 compared to the benign lesion and healthy control groups (P < 0.05). The AUCs of the three markers for diagnosing cervical cancer were 0.8338, 0.8379, and 0.8466, respectively (P < 0.0001). Tumor marker levels showed an increasing trend with advancing TNM staging and decreasing differentiation degree (P < 0.05). Patients with squamous cell carcinoma had significantly higher SCC-Ag levels, while those with adenocarcinoma had significantly higher CEA and CA125 levels (P < 0.05). At 2-year follow-up, the recurrence group exhibited significantly higher tumor marker levels before treatment compared to the non-recurrence group (P < 0.05).

**Conclusion:**

Serum levels of SCC-Ag, CEA, and CA125 are closely associated with the pathological characteristics of cervical cancer, demonstrating high diagnostic value. Pre-treatment tumor markers levels serve as important reference indicators for prognostic assessment.

## Introduction

Cervical cancer is one of the most common malignant tumors of the female reproductive system, ranking fourth in incidence and fourth in mortality among female malignancies worldwide (Sahasrabuddhe, 2024). According to the World Health Organization, approximately 600,000 new cases of cervical cancer are diagnosed globally each year, with over 340,000 deaths. In China, cervical cancer ranks sixth in incidence and seventh in mortality among female malignancies, exhibiting a trend toward younger age groups and posing a serious threat to women’s health and life safety (Voelker, 2023; Poddar and Maheshwari, 2021). Currently, the diagnosis of cervical cancer primarily relies on cervical cytology screening, HPV testing, colposcopy, and histopathological examination. Although these approaches play a crucial role in cervical cancer screening and diagnosis, they still have certain limitations, such as false-negatives in cytology screening, invasiveness of colposcopy, and possible sampling errors in pathological biopsy (Gopu et al., 2021; Lizano et al., 2024). Therefore, identifying simple, non-invasive, and reproducible auxiliary diagnostic indicators is of great significance for the clinical management of cervical cancer.

Serum tumor marker testing is widely used in the screening, diagnosis, efficacy evaluation, and prognosis assessment of malignant tumors due to its advantages of easy sampling, minimal invasiveness, and dynamic monitoring. Squamous cell carcinoma antigen (SCC-Ag) is a specific marker for squamous cell carcinoma and is often significantly elevated in patients with cervical squamous cell carcinoma. Carcinoembryonic antigen (CEA), as a broad-spectrum tumor marker, can be elevated in various malignant tumors. Carbohydrate antigen 125 (CA125) is primarily used for the diagnosis and monitoring of ovarian cancer, but it may also be elevated in patients with cervical cancer, particularly adenocarcinoma (Jha et al., 2023). A previous study (Lycke et al., 2023) has indicated that the levels of these tumor markers are correlated with pathological characteristics of cervical cancer, such as clinical staging, pathological type, and lymph node metastasis, although the findings have varied among different studies. In addition, the prognosis for cervical cancer patients is affected by multiple factors, including clinical staging, pathological type, degree of differentiation, lymph node metastasis status, etc. Despite ongoing improvements in treatment methods for cervical cancer, including surgery, radiotherapy, chemotherapy, and targeted therapy, some patients still experience recurrence and metastasis, and the five-year survival rate remains to be improved (Adiga et al., 2021). Therefore, identifying reliable prognostic indicators can help recognize high-risk patients, develop personalized treatment plans, and improve patient outcomes.

Based on the above background, this study aimed to retrospectively analyze the serum SCC-Ag, CEA, and CA125 levels in patients with cervical cancer, patients with benign cervical lesions, and healthy controls, explore the diagnostic value of these tumor markers for cervical cancer, analyze their association with pathological characteristics, and evaluate the predictive effect of pre-treatment tumor marker levels on prognosis, thereby providing a reference for the clinical diagnosis and treatment of cervical cancer.

## Materials and methods

### Study population

This retrospective study was approved by the ethics committee of Zhuhai Hospital of Integrated Traditional Chinese and Western Medicine and complied with the ethical requirements of the Declaration of Helsinki. Clinical data were collected from the hospital information system between January 2022 and June 2023. A total of 82 patients with cervical cancer admitted to the gynecology department of our hospital were assigned to the cervical cancer group (CG), 98 patients with benign cervical lesions to the benign lesion group (BG), and 80 healthy women from the health examination center to the healthy control group (HG).

Inclusion criteria for the cervical cancer group: (1) Histopathologically confirmed as cervical cancer; (2) Newly diagnosed patients who had not received any anti-tumor treatment; (3) Complete clinical data, including pathological type, TNM staging, and degree of differentiation; (4) Patients who had completed serum tumor marker testing before surgery; (5) Complete follow-up data. Exclusion criteria: (1) Presence with other malignant tumors; (2) Severe cardiac, hepatic, or renal insufficiency; (3) Autoimmune diseases; (4) Pregnant or lactating women.

Inclusion criteria for the benign lesion group: (1) Pathologically confirmed benign cervical lesions, including chronic cervicitis, cervical polyps, cervical myomas, etc.; (2) No history of malignant tumors; (3) Completion of serum tumor marker testing.

Inclusion criteria for healthy control group: (1) Healthy women with normal physical examination results; (2) No history of cervical disease; (3) No history of malignant tumors; (4) Normal cervical cytology examination.

### Study methods

#### Collection of general information

Basic information of all study subjects was collected, including age, body mass index, menstrual history, reproductive history, and smoking history. Pathological data such as TNM staging (according to the International Federation of Gynecology and Obstetrics 2018 staging criteria (Salib et al., 2018)), pathological type (squamous cell carcinoma, adenocarcinoma), degree of differentiation (well, moderately, poorly differentiated), and lymph node metastasis were also collected for patients with cervical cancer.

#### Serum tumor marker testing

Serum levels of SCC-Ag, CEA, and CA125 were collected from laboratory tests of study subjects (using electrochemiluminescence immunoassay).

#### Follow-up status

All cervical cancer patients underwent regular follow-up after discharge. Follow-up procedures included gynecological examinations, tumor marker testing, and imaging studies. The time of recurrence, site of recurrence, and survival status were recorded. The follow-up period concluded in June 2024.

### Statistical analysis

Data analysis was performed using SPSS 26.0 statistical software. Continuous data conformed to normal distribution following Shapiro-Wilk test and were expressed as mean ± standard deviation. Comparisons between two groups were conducted using the independent samples t-test, while comparisons among multiple groups were performed using one-way analysis of variance (ANOVA). Pairwise comparisons between groups were performed using the LSD-t test. Categorical data were expressed as number of cases [n (%)], and comparisons between groups were performed using the chi-square test. The receiver operating characteristic (ROC) curves were used to evaluate the diagnostic value of tumor markers for cervical cancer, and the area under the curve (AUC), sensitivity, and specificity were calculated. The difference was considered statistically significant when P < 0.05.

## Results

### Analysis of baseline clinical data for study subjects

This study included 82 cervical cancer patients, 98 patients with benign cervical lesions, and 80 healthy controls between January 2022 and June 2024. There was no statistically significant difference in age among the three groups (P > 0.05) (Table 1).

**TABLE 1:** Analysis of baseline clinical data for study subjects (mean ± SD)/[n (%)].

Clinical characteristics	Cervical cancer group (n = 82)	Benign lesion group (n = 98)	Healthy control group (n = 80)
Age (years)	52.45 ± 10.78	49.23 ± 11.26	48.67 ± 10.45
TNM staging			
Stage I	35 (42.68)	-	-
Stage II	28 (34.15)	-	-
Stage III	15 (18.29)	-	-
Stage IV	4 (4.88)	-	-
Pathological type			
Squamous cell carcinoma	67 (81.71)	-	-
Adenocarcinoma	15 (18.29)	-	-
Degree of differentiation			
Well differentiated	22 (26.83)	-	-
Moderately differentiated	38 (46.34)	-	-
Poorly differentiated	22 (26.83)	-	-
Lymph node metastasis			
Positive	27 (32.93)	-	-
Negative	55 (67.07)	-	-

### Comparison of serum tumor marker levels among the three groups

**FIGURE 1:**
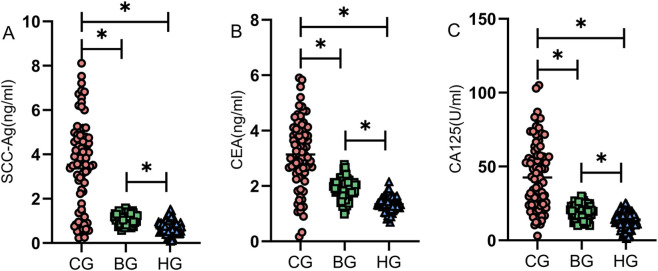
Comparison of serum tumor marker levels among the three groups. The cervical cancer group demonstrated significantly higher levels of SCC-Ag **(A)**, CEA **(B)**, and CA125 **(C)** compared to the benign lesion and healthy control groups (P < 0.05). Note: * indicates statistically significant differences between groups.

The serum levels of tumor markers SCC-Ag, CEA, and CA125 were collected from the three groups and compared for intergroup differences. Results indicated that the cervical cancer group demonstrated significantly higher tumor marker levels compared to the benign lesion and healthy control groups (P < 0.05) (Figure 1).

### Analysis of the diagnostic value of tumor markers for cervical cancer

**FIGURE 2:**
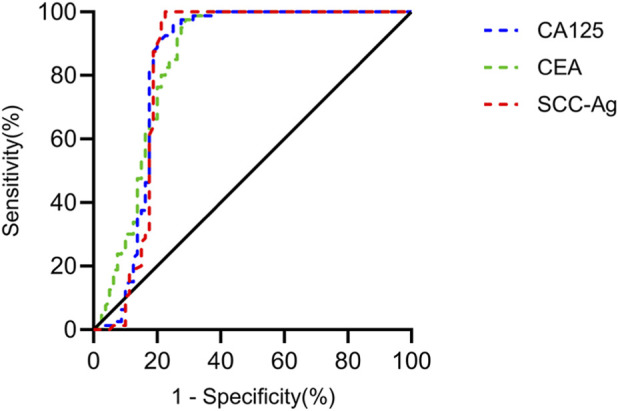
Analysis of the diagnostic value of tumor markers for cervical cancer The AUCs for SCC-Ag, CEA, and CA125 in diagnosing cervical cancer were 0.8338, 0.8379, and 0.8466, respectively (P < 0.0001).

SCC-Ag, CEA, and CA125 were used to diagnose cervical cancer. The calculated diagnostic AUCs for these markers were 0.8338, 0.8379, and 0.8466, respectively (P < 0.0001) (Table 2; Figure 2).

**TABLE 2:** Analysis of the diagnostic value of tumor markers for cervical cancer.

Diagnostic indicator	AUC	SE	95% CI	P	Sensitivity	Specificity
SCC-Ag	0.8338	0.0390	0.7573-0.9102	<0.0001	87.50	81.25
CEA	0.8379	0.0376	0.7641-0.9117	<0.0001	81.25	82.50
CA125	0.8466	0.0339	0.7801-0.9131	<0.0001	80.00	78.75

### Differences in tumor marker levels among cervical cancer patients with different TNM stages

**FIGURE 3:**
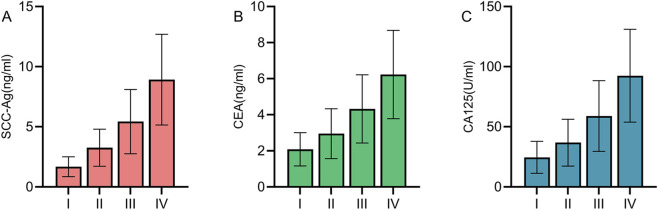
Differences in tumor marker levels among cervical cancer patients with different TNM stages As TNM staging progressed, the levels of SCC-Ag **(A)**, CEA **(B)**, and CA125 **(C)** showed an increasing trend (P < 0.05).

Patients included in the study were grouped and compared according to TNM staging. There were 35 patients in Stage I, 28 patients in Stage II, 15 patients in Stage III, and 4 patients in Stage IV. The comparison revealed that as TNM staging progressed, the levels of the three cervical cancer tumor markers showed an increasing trend (P < 0.05) (Figure 3).

### Comparison of tumor marker levels among cervical cancer patients with different degrees of differentiation

**FIGURE 4:**
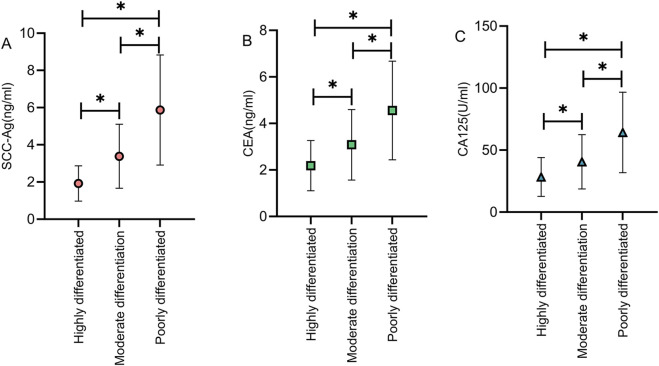
Comparison of tumor marker levels among cervical cancer patients with different degrees of differentiation. As the degree of tumor differentiation decreased, the levels of tumor markers SCC-Ag **(A)**, CEA **(B)**, and CA125 **(C)** show an increasing trend (P < 0.05). Note: * indicates statistically significant differences between groups.

The included cervical cancer patients were categorized into well-differentiated (n = 22), moderately differentiated (n = 38), and poorly differentiated (n = 22) groups according to the degree of differentiation. Comparison of tumor marker levels among the three groups revealed a significant upward trend in marker levels as tumor differentiation decreased (P < 0.05) (Figure 4).

### Comparison of tumor marker levels among cervical cancer patients with different pathological types

**FIGURE 5:**
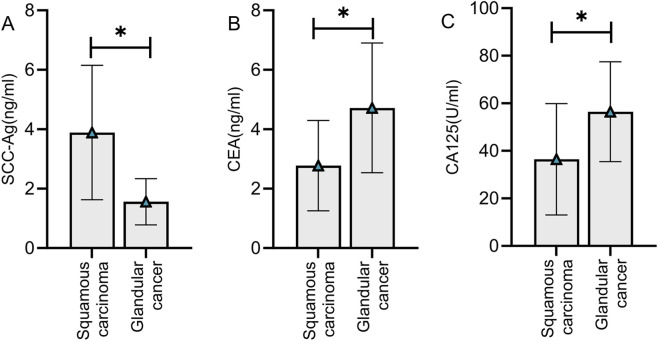
Comparison of tumor marker levels among cervical cancer patients with different pathological types. Patients with squamous cell carcinoma had higher SCC-Ag levels **(A)** than those with adenocarcinoma, whereas patients with adenocarcinoma showed higher CEA **(B)** and CA125 **(C)** levels than those with squamous cell carcinoma (P < 0.05). Note: * indicates statistically significant differences between groups.

The included cervical cancer patients were categorized into squamous cell carcinoma group (n = 67) and adenocarcinoma group (n = 15). Comparative analysis revealed that patients with squamous cell carcinoma had higher SCC-Ag levels than those with adenocarcinoma, whereas patients with adenocarcinoma showed higher CEA and CA125 levels than those with squamous cell carcinoma (P < 0.05) (Figure 5).

### Comparison of tumor marker levels before treatment among cervical cancer patients with different prognoses

**FIGURE 6:**
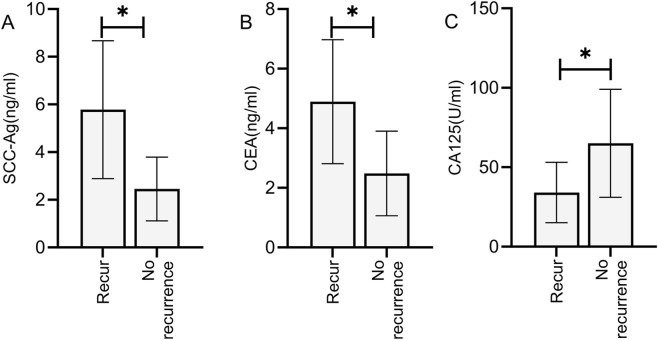
Comparison of tumor marker levels before treatment among cervical cancer patients with different prognoses. Patients in the recurrence group exhibited significantly higher pre-treatment levels of SCC-Ag **(A)**, CEA **(B)**, and CA125 **(C)** compared to the non-recurrence group (P < 0.05). Note: * indicates statistically significant differences between groups.

Based on 2-year follow-up results, the included patients were categorized into a recurrence group (n = 63) and a non-recurrence group (n = 19). Comparison revealed that the recurrence group showed significantly higher pre-treatment tumor marker levels compared to the non-recurrence group (P < 0.05) (Figure 6).

## Discussion

### Value of serum tumor markers in cervical cancer diagnosis

The results of this study indicated that the cervical cancer group demonstrated significantly higher serum levels of SCC-Ag, CEA, and CA125 compared to the benign lesion and healthy control groups, suggesting abnormal expression of these tumor markers in cervical cancer patients. Further ROC curve analysis revealed that the AUCs for all three biomarkers in diagnosing cervical cancer exceeded 0.83, indicating their high diagnostic value. Among them, CA125 demonstrated the highest AUC value (0.8466), followed by CEA (0.8379) and SCC-Ag (0.8338). A previous study (Wu et al., 2021) indicated that SCC-Ag is a glycoprotein produced by squamous epithelial cells, present at extremely low levels in normal squamous epithelium, but significantly elevated during malignant transformation. The results of this study indicated that SCC-Ag demonstrated sensitivity and specificity exceeding 80% for cervical cancer diagnosis, consistent with findings from previous studies (Ye et al., 2020; Guo et al., 2020). CEA is a clinically widely used broad-spectrum tumor marker that can be elevated in various malignant tumors such as colorectal cancer, lung cancer, breast cancer, and gastric cancer. Its non-specific characteristics determine that CEA is not suitable as an independent diagnostic indicator for cervical cancer and should be used in combination with more specific markers such as SCC-Ag. The results of the present study demonstrated that CEA levels were more significantly elevated in cervical adenocarcinoma. This differential expression pattern may have certain reference value in distinguishing pathological types (Zhang et al., 2020). The results of this study indicated that CEA demonstrated sensitivity and specificity exceeding 80% for cervical cancer diagnosis, suggesting its potential as an auxiliary diagnostic marker for cervical cancer. CA125 is primarily expressed in peritoneal mesothelial cells and Müllerian epithelial cells. Cervical adenocarcinoma originates from the glandular epithelium of the cervical canal, sharing a similar embryonic origin with Müllerian epithelium. Consequently, elevated CA125 levels are observed in cervical adenocarcinoma (Bălăceanu et al., 2025). Although the aforementioned tumor markers all demonstrate good diagnostic efficacy for cervical cancer, the diagnostic performance of a single marker may be limited. Combined diagnosis can enhance diagnostic accuracy. Future efforts will focus on establishing diagnostic models incorporating multiple markers, integrating them with patient clinical characteristics, and developing a cervical cancer risk prediction scoring system to provide more precise guidance for clinical decision-making. Although various tumor markers show moderate to good diagnostic performance, their sensitivity and specificity remain insufficient to support their application as independent diagnostic tools. These markers should be regarded as auxiliary means to supplement rather than replace standard diagnostic methods such as cervical cytology, HPV testing, and histopathological examination. Future research may explore whether the combined detection of multiple markers or their integration with clinical parameters can further improve diagnostic accuracy.

### Relationship between tumor marker levels and pathological characteristics

This study found that serum tumor marker levels were significantly correlated with multiple pathological characteristics of cervical cancer. First, as TNM staging progresses, the levels of SCC-Ag, CEA, and CA125 showed an increasing trend. Marker levels were highest in stage IV patients and lowest in stage I patients. This finding suggests that tumor marker levels can reflect the degree of tumor progression. Patients with advanced cervical cancer have larger tumors and an increased number of tumor cells, which in turn leads to an elevation in the secretion of tumor-associated antigens and an increase in serum marker levels. Furthermore, previous studies (Kim et al., 2021; Kakimoto et al., 2021) indicate that advanced tumors are often accompanied by local invasion and distant metastasis, with increased metabolic activity of tumor cells potentially promoting the production and release of biomarkers. The findings of this study also revealed a negative correlation between tumor differentiation and biomarker levels, with lower degrees of differentiation associated with higher biomarker levels. Previous research (Aydos et al., 2023) indicates that poorly differentiated tumors exhibit high malignancy, marked cellular atypia, rapid proliferation, and strong invasive capacity. These biological characteristics may lead to the overexpression of tumor markers. In this study, patients in the poorly-to-moderately differentiated group exhibited higher levels of all three tumor markers compared to those in the well-differentiated group. This suggests that tumor markers may serve as reference indicators for assessing tumor malignancy. Finally, the study compared tumor marker levels among cervical cancer patients with different types of cervical cancer. The results indicated that SCC-Ag levels were higher in squamous cell carcinoma patients than in adenocarcinoma patients, while CEA and CA125 levels were higher in adenocarcinoma patients than in squamous cell carcinoma patients. The aforementioned phenomena are attributable to the tissue origins and biological characteristics of different pathological types. SCC-Ag is specifically expressed in squamous epithelial cells, resulting in more pronounced elevation in squamous cell carcinoma. Conversely, CEA and CA125 exhibit higher expression in tumors of glandular epithelial origin, reflecting the secretory nature of adenocarcinomas (Huang et al., 2020; Xiao et al., 2024). This finding suggests that the expression pattern of tumor markers may help determine the pathological type of cervical cancer, providing a reference for clinical diagnosis.

### Prognostic value of tumor markers

Prognosis assessment is a critical component in the clinical management of cervical cancer, aiding in the development of personalized treatment strategies and follow-up plans. Through a 2-year follow-up, this study found that patients in the recurrence group had significantly higher pre-treatment levels of SCC-Ag, CEA, and CA125 compared to the non-recurrence group, suggesting that pre-treatment tumor marker levels may serve as important prognostic predictors. This phenomenon may be attributed to the following reasons: Elevated tumor marker levels indicate a higher tumor burden and greater tumor activity. These patients may have micrometastases or circulating tumor cells, resulting in a high risk of recurrence even after radical treatment (Yokota et al., 2023). Previous research (Tony et al., 2023) demonstrates that SCC-Ag levels are negatively correlated with disease-free survival and overall survival in cervical cancer patients, with those exhibiting higher pre-treatment SCC-Ag levels showing poorer prognosis. This finding aligns with the results of the present study, which supports this perspective and further confirms that CEA and CA125 also possess comparable prognostic predictive value. Based on the above findings, patients with elevated tumor marker levels prior to treatment may require more aggressive therapeutic strategies, such as expanding surgical scope, intensifying adjuvant therapy, or shortening follow-up intervals. Concurrently, dynamic monitoring of tumor marker changes facilitates early detection of recurrence and timely adjustment of treatment regimens (Che et al., 2022; Sottotetti et al., 2022). However, tumor markers are not definitive prognostic indicators and require comprehensive evaluation in conjunction with other clinical and pathological factors. In the future, a multifactorial prognostic prediction model incorporating tumor markers may be established to improve the accuracy of prognosis assessment.

## Clinical significance, limitations, and future research directions

The findings of this study provide important guidance for the clinical management of cervical cancer. First, serum tumor marker testing can serve as an auxiliary means of cervical cancer screening and diagnosis, particularly when cytology and HPV test results are inconclusive. Tumor marker levels can provide additional diagnostic information. Second, the correlation between tumor marker levels and pathological characteristics aids in preoperative assessment of tumor malignancy and progression, providing a reference for formulating surgical plans and predicting surgical difficulty. Third, pre-treatment tumor marker levels can be used for prognostic stratification, identifying high-risk patients, and guiding personalized treatment. Finally, serum tumor marker testing may be used as a supplementary reference indicator for comprehensive clinical assessments. In combination with cytological examination, HPV testing, and colposcopy results, it can provide additional information for clinical decision-making but cannot replace existing standard screening and diagnostic methods.

Although this study provides valuable insights for the early diagnosis and prognostic assessment of cervical cancer, it also has certain limitations. Firstly, as a retrospective study, it may be subject to selection bias and information bias, which could affect the reliability of the results. Secondly, the relatively limited sample size in this study may affect the statistical power of subgroup analyses, especially comparisons of subgroups with different TNM stages, differentiation degrees, and pathological types. Finally, the follow-up period was relatively short, and long-term prognosis requires further observation. To address the aforementioned limitations, future prospective, multicenter, large-sample research will be conducted to validate the generalizability of these findings. In addition, increasing sample size and extending follow-up duration may further validate the clinical recommendations proposed in this study.

The serum levels of SCC-Ag, CEA, and CA125 are significantly elevated in cervical cancer patients, demonstrating high diagnostic value for cervical cancer. Tumor marker levels are closely associated with pathological characteristics such as TNM staging, differentiation degree, and pathological type, and can reflect the biological behavior of tumors. Elevated tumor marker levels prior to treatment indicate a poor prognosis and serve as a critical reference indicator for prognostic assessment. Serum tumor marker testing facilitates early diagnosis, disease assessment, and prognosis evaluation of cervical cancer; however, large-scale, multicenter, prospective studies are still required to further validate its clinical utility.

## Data Availability

The original contributions presented in the study are included in the article/supplementary material, further inquiries can be directed to the corresponding author.
